# Sex-biased parasitism in vector-borne disease: Vector preference?

**DOI:** 10.1371/journal.pone.0216360

**Published:** 2019-05-02

**Authors:** Camille-Sophie Cozzarolo, Nicolas Sironi, Olivier Glaizot, Romain Pigeault, Philippe Christe

**Affiliations:** 1 Département d’Ecologie & Evolution, Université de Lausanne, Lausanne, Suisse; 2 Musée cantonal de zoologie, Lausanne, Suisse; Instituto Nacional de Salud Pública, MEXICO

## Abstract

Sex-biased infections are a recurrent observation in vertebrates. In many species, males are more parasitized than females. Two potentially complementary mechanisms are often suggested to explain this pattern: sexual differences in susceptibility mainly caused by the effect of sex hormones on immunity and differential exposure to parasites. Exposure is mostly a consequence of host behavioural traits, but vector-borne parasitic infections involve another degree of complexity due to the active role of vectors in transmission. Blood-sucking insects may make choices based on cues produced by hosts. Regarding malaria, several studies highlighted a male-biased infection by *Plasmodium* sp in great tits (*Parus major*). We hypothesize that the mosquito vector, *Culex pipiens*, might at least partially cause this bias by being more attracted to male birds. Intrinsic variation associated to bird sex would explain a preference of mosquitoes for males. To test this hypothesis, we provide uninfected mosquitoes with a choice between uninfected male and female nestlings. Mosquito choice is assessed by sex typing of the ingested blood. We did not observe any preference for a given sex. This result does not support our prediction of a preference of mosquitoes for male great tits during the nestling period. In conclusion, mosquitoes do not seem to have an intrinsic preference for male nestlings. However, sexually divergent traits (*e*.*g*. behaviour, odour, metabolic rate) present in adults may play a role in the attraction of mosquitoes and should be investigated.

## Introduction

Prevalence and intensity of parasitism in vertebrates, is often higher in males than females ([1–6] but see [7]). Sex differences in susceptibility, development and exposure (reviewed in [8]) can explain sex-biased infection. Firstly, male-biased parasitism may be due to how parasites perform in each sex. Sex hormones have different influences on the immune system. Both androgens and oestrogens suppress cell-mediated immunity, but oestrogens can stimulate humoral immunity [9]. In particular, some studies on birds show evidence for the immunosuppressive role of testosterone ([10–12] but see [13, 14]). From this perspective, we might expect parasites to perform better within a male host.

Secondly, sex differences in behaviours linked to reproduction [15, 16], foraging [17] or social status [18, 19] may also induce a sex-biased exposition to parasite. For example, a reduction of grooming activities at the expense of time spent for territorial defences during the breeding season may imply that males harbour higher ectoparasite load than females as showed in impalas infected by ticks [20]. In contrast, females might be more exposed than males if they aggregated in nursery colonies, as shown for example in different bat species [7].

In vector-borne parasite diseases, vectors add another level of complexity. For instance, blood-feeding invertebrates such as mosquitoes actively seek for a blood meal and may differentially encounter male or female vertebrate hosts. In that case, sex-biased parasitism will result from specific host/vector interaction [21, 22]. In addition, mosquitoes mostly use olfaction during host-seeking and volatile organic compounds (VOC) and CO2 are crucial cues for them [23–25]. Because males are often bigger than females in both mammals and birds, they produce more CO2 [26–28] and may therefore be more easily detected by vectors. In addition, males’ odours are known to differ from females’ [29–32]. Both factors may result into a higher attractiveness of males to mosquitoes. Wild caught mosquitoes were found to feed more on male birds (64.0%) than females (36.0%, of 308 samples), consistently across mosquito species [33].

Other factors may influence mosquitoes’ host-choice, as for example relative humidity, parasitic infection, defensive behaviours or body heat (reviewed in [34]). In particular, mosquitoes are attracted to heat [35–38] or heated baits [39, 40]. However, this cue seems to act on mosquito behaviour at close proximity to the host mainly [41]. Former studies on different bird species found that females have a slightly higher temperature than males in most cases [42, 43], but it is not known whether it can influence mosquito choices.

*Plasmodium* is a mosquito-borne haemosporidian parasite that infects many different vertebrate host species. A male biased infection was observed in many host / parasite pairs [16, 44–48]. Differential exposure to mosquito bites between sexes may thus explain the observed sex-biased infection.

In this study, we investigated the role of *Culex pipiens*, the natural mosquito vector of *Plasmodium* in great tits in sex-biased malaria infection. Indeed, male-biased infection in great tits was observed in several studies [16, 49, 50]. A study of 13 years on two natural populations of *P*. *major* also found a sex effect on *Plasmodium* infection prevalence, with males being more infected than females (S1 Table). In the present study, we tested whether juvenile male birds were more attractive to mosquitoes by placing 18 pairs composed of a male and a female chick coming from a same nest in a 1-meter-long box together with 25 mosquitoes for one hour and by identifying the sex of the host of each mosquito by PCR.

## Materials and methods

### Ethical statement

This experiment was approved by the Ethical Committee of the Vaud Canton veterinary authorities, licence number 1730.4. Birds were caught and ringed under licence with the permit number F044-0799 of the Swiss Federal Office for the Environment.

### Experimental system

#### Great tit collection and rearing

A total of 38 Great tits (*P*. *major*) were used during this study. Hatching date of new-born nestlings was determined by monitoring nest boxes in three different study sites: Dorigny (46°31’26”N; 6°34’48”E; alt. 409 m), Monod (46°34’27”N; 6°23’32”E; alt. 686 m) and La Praz (46°40’12”N; 6°25’06”E, 1032m) from March to June. Three to five days after hatching (day 0), the nests were microwaved to eliminate all ectoparasites. Nestlings aged between six and nine days were blood sampled to determine their sex and infection status and ringed with an individual metallic ring. Five to 10μL of blood were sampled from the medial metatarsal vein and stored in PBS at 4°C until DNA extraction and molecular sexing (see Molecular analyses). At day 14, nestlings were weighted (electronic scales ± 0.1 g), tarsus and wing length were measured using an electronic metal calliper (± 0.01mm).

#### Mosquito rearing

We used a *Culex pipiens* lab colony, issued from egg rafts sampled in September 2016 in the Dorigny forest. Mosquitoes were reared in an insectary at 25°C ± 1°C, 70 ± 5% RH and with 12L:12D photoperiod. On the hatching day, larvae were seeded into plastic trays containing 500 mL of mineral water (Fonte Tavina Naturale, Italia) at a constant density (130 ± 5 individuals per tray). Larvae were fed ad libitum every two days until pupation with a mixture composed by TetraMin Junior fish pellets, Schweizer Classic rabbit pellets and JBL Novo Malawi fish flakes (1:1:1 ratio). Tray water was changed every 3 days. On pupation day, plastic trays provided with 10% sugar solution were placed in emergence cages. Experimentally naïve female were deprived of sugar solution 20 h prior to the experiment, in order to maximize the biting rate. Water was provided from 20 h to 6 h before the experiment to prevent dehydration.

#### Experimental procedure

Host choice behaviour experiments were performed by simultaneously presenting an average of 25 (range: 20–30) female mosquitoes to a 14 days old male and female great tit. To reduce individual and family effects all couples of nestlings were formed by a male and a female coming from the same nest and were of similar weight. We chose to perform this experiment with 14-day-old nestlings (as in [51]) because their tarsus length have reached their final size at this age. If they are disturbed when they are older (more than 16 days old), this may provoke early fledging. We also estimated that at this age, they had not fully acquired anti-ectoparasite behaviours, so we would not need to consider this parameter. Moreover, when 14 days old, haemosporidian parasites were never detected in their blood. Because infection status may influence mosquito biting choice [52, 53], performing the experiment at this stage allows avoiding this confounding factor.

Birds’ cloacal body temperature were taken 30 minutes before the assay then each bird was placed in the cup at one of the extremities of a rectangular, transparent Plexiglas box (100cm x 20cm x 20cm, 0.6 cm thickness, see S1 Fig). An ambient air flow was created by an axial flow propeller pump, and ambient air was pumped inside the box from the two sides. Incoming airflow (20 ± 1 cm/s) was controlled on both sides using a hot-wire anemometer. Mosquitoes were released in the middle of the box in a section closed by two mosquito-net screens and were allowed to settle for 5min before the start of the experiment. Then the screens were removed and the mosquitoes were given the opportunity to select and bite one of the birds. The experiments lasted for one hour and fed mosquitoes were then collected and stored at -80°C. After each trial, all the material was cleaned using 96% ethanol and then air dried.

### Molecular analyses

For the mosquito choice experiments, DNA was extracted from the blood meal of mosquitoes using a Qiagen BioSprint 96 workstation following the tissue protocol for extraction (Qiagen, Hilden, Germany). A PCR was used to assess sex of great tit nestlings at the beginning of the study and to assess the host upon which mosquitoes fed during the choice experiment. We used three primers targeting CHD1, a gene located on birds’ sex chromosome that contains an intron with a constant size difference between W and Z chromosomes (2987 F, 3007 F, 3112 R, [54]). Sex was determined by examination of the agarose gel (2%) after electrophoresis, where migrated samples of female DNA produced two bands, while males only one [54]. A nested PCR [55] followed by an electrophoresis on agarose gel (2%) was performed to detect haemosporidian (*Plasmodium*, *Haemoproteus* and *Leucocytozoon*) infection status in birds, in order to ensure that we were testing only uninfected nestlings.

### Statistical analyses

All analyses were performed in *R* 3.3.2. The effect of sex on bird attractiveness was tested using a repeated G-test of goodness of fit [53, 56]. The observed preference for male great tits (proportion of mosquitoes fed on the male relative to the total number of blood-fed mosquitoes) was tested against the predicted no-choice value of *p* = 0.5. We also performed the repeated G-test of goodness of fit on subsets of the data, separating the trials according to the site of origin of the birds, as well as on the total data, pooling the trials per site, in order to check whether the proportion of mosquitoes biting the male bird would differ according to the origin of the pair of birds. To assess whether other variables had an effect on bird attractiveness, the proportion of mosquitoes fed on male birds was analysed using Generalized Linear Mixed Models (GLMM) with a binomial error distribution [57]. Models were fitted by specifying the differences in body mass and temperature as fixed effects. The date of the experiment and the origin (site) were included as random effects. Model selection was performed by stepwise elimination of variables that were not significant (*p* > 0.05) by Likelihood Ratio Tests (LRT), using a *χ*^2^ test to estimate the significance (“lme4” package, [57]). In previous studies with *Cx pipiens*, the percentage of mosquitoes fed with a mix of blood (mosquitoes fed on more than one bird) reached up to 10% of fed mosquitoes in trials lasting 12 hours [58] and 3–6.2% in trials lasted two hours [53, 56]. In our experiment mixed blood meal would overlap in the agarose gel and appear as a female blood meal. Therefore, although our trials lasting only 1 hour, we ran a conservative parallel analysis on data where 10% of mosquitoes fed on females were excluded, to simulate the exclusion of potential mixed blood meals.

## Results

None of the tested nestlings were infected with haemosporidian parasite. Eighteen trials were performed and the blood meal origin of 387 mosquitoes was identified. The mean (± SE) number of mosquitoes that fed per trial was 21.5 ± 1.68 (81% ± 0.04 SE, range: 35%-100%). The outcome of each trial is reported in (S2 Table).

A mean of 47.9% ± 0.03 SE of fed mosquitoes took blood from male nestlings (range: 31.8%-69%). Nestling sex did not affect the mosquito feeding preference, as the replicated G-test of goodness of fit did not show statistically significant departure from the proportion in the absence of choice (total-G = 27.64, 18df, p = 0.068; pooled-G = 1.14, 1df, p = 0.286, heterogeneity-G = 26.50, 17df, p = 0.066). Similar results were obtained in statistical analyses with data where 10% of mosquitoes fed on females were excluded (total-G = 25.116, 18df, p = 0.092; pooled-G = 0.001, 1df, p = 0.975, heterogeneity-G = 25.115, 17df, p = 0.0922). When separating the data according to the site of origin of the birds, there was also no significant departure from the no-choice proportion for any of the site, and there was no significant difference in the proportion of mosquitoes biting the male bird when comparing the sites of origin of the birds (S3 Table).

**Fig 1:**
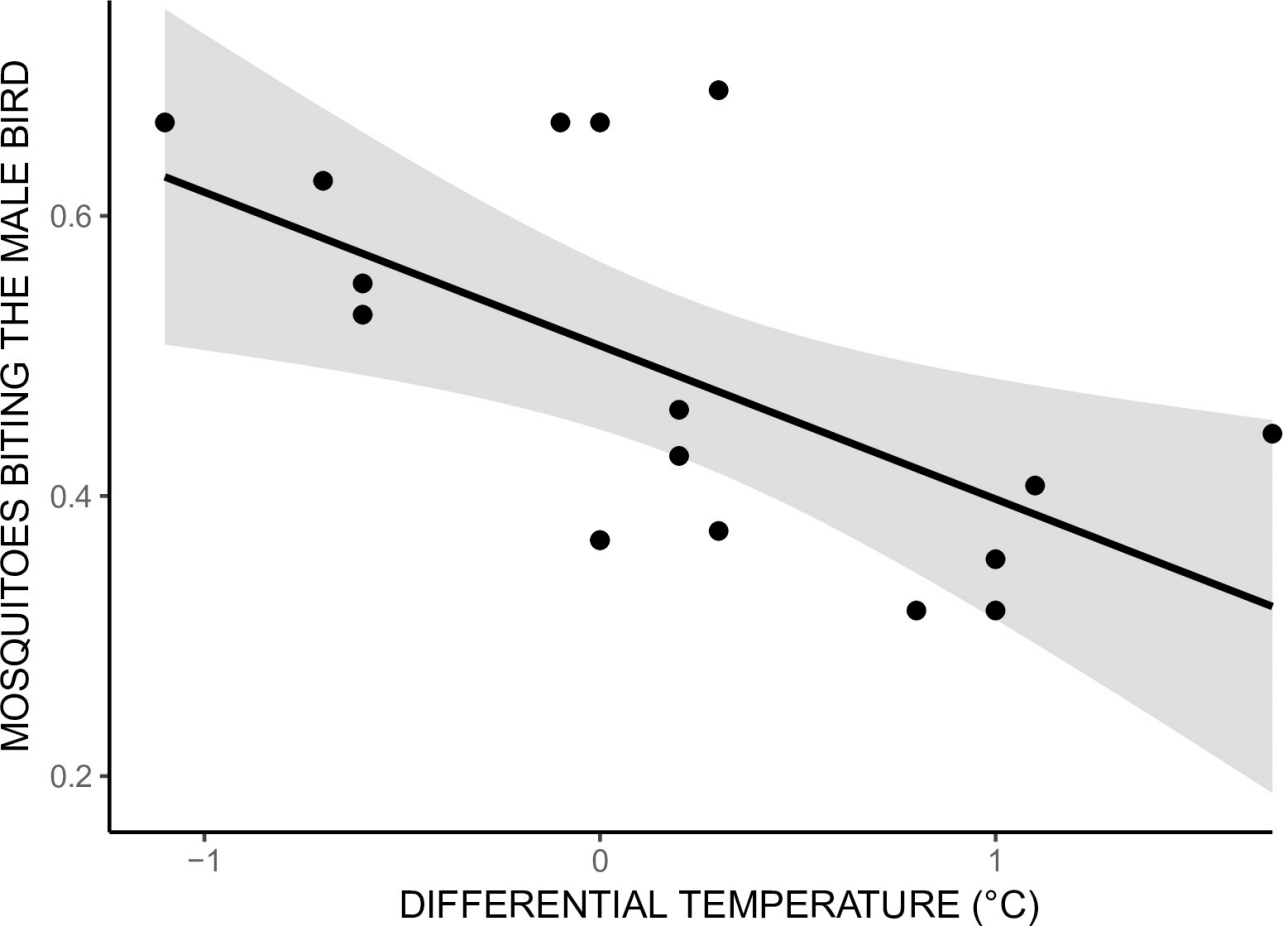
Proportion of mosquitoes biting the male in function of the difference in temperature. Each dot represents a trial in which a pair composed two nestlings (brother and sister) was exposed to mosquito bites. The x-axis represents the male nestling’s temperature minus the female nestling’s temperature. The y-axis represents the proportion of mosquitoes that have bitten the male bird.

The difference in bird temperature significantly influenced mosquitoes’ choice (*χ*^2^ = 6.969, p = 0.008). Individuals with lower temperature were preferentially chosen independently of their sex (Fig 1). There was no effect of body weight (*χ*^2^ = 1.166, p = 0.280) on mosquitoes’ choice. Results were similar when 10% of mosquitoes fed on females were excluded to correct for potential bias due to mixed blood meals (body temperature: *χ*^2^ = 7.864, p = 0.005, body weight *χ*^2^ = 1.110, p = 0.292).

## Discussion

In this study, we tested whether mosquitoes bite preferentially juvenile male great tits. We found no preference for one of the sexes in great tit nestlings. These results suggest that the sex-biased infection observed in natural great tits populations [S1 Table] was not caused by an intrinsic preference of mosquitoes for juvenile male birds.

There is evidence that vertebrate hosts vary in their attractiveness to mosquitoes [59] and that host-vector contact is far from random [60]. Indeed, the composition of the host’s odour profile and its CO_2_ emission intensity may influence mosquitoes’ choice for specific host profile [61, 62]. Traits such as odour and metabolic rates are predicted to differ more between sexes in sexually mature birds than in nestlings. In zebra finches, no difference in metabolic rates between male and female nestlings was reported [63], but adult females had a higher metabolic rate than males [64, 65]. A difference was also observed in adult great tits [28]. Nevertheless, in this species the adult males have higher metabolic rate than females [28]. Regarding odour, nestlings of both sexes live together in a closed environment and therefore their odours probably mix. In addition, the uropygial gland, involved in volatile organic compounds emission [31, 66, 67], is not yet fully functional in nestlings [68, 69]. Conversely, in adult birds, several studies reported a difference in odour profile between sexes [31, 70–73]. The absence of a sex-based preference in mosquitoes in our experiment might therefore be due to the use of nestlings which are not sexually mature and probably do not differ strongly in their odour profile. Using adult great tits to test the effect of *Plasmodium* infection on attraction of *Cx pipiens*, [52] found a marginally higher attractiveness of females.

A recent study showed that house sparrows with lower metabolic rate suffered more mosquito bites than individuals with higher metabolic rate [58]. This result is surprising because a higher metabolic rate is expected to be associated with higher CO_2_ emission and therefore should induce a higher attractiveness [58, 74]. However, a higher metabolic rate might also result from a high activity level [58, 75]. At the time of biting, mosquitoes tend to avoid individuals that are more active [58, 76]. Indeed, the defensive behaviour displayed by birds may reduce the ability of mosquitoes to take a blood meal. Interestingly we also observed that birds with a lower body temperature were preferentially chosen regardless of their sex. Although mosquitoes are attracted to heat when host seeking [35, 36, 38], to our knowledge, there was previously no evidence that mosquitoes choose the warmer or colder host, when they have the choice. The negative association between mosquito feeding preference and bird body temperature could be explained by the fact that mosquitoes avoid active individuals [58, 76], which may have a higher body temperature due to their activity [77, 78].

Our study leaves the question of why a male-biased infection exists in *P*. *major* unanswered. Several life-history traits of birds could be involved. Firstly, male great tits may be more infected due to a higher exposure to mosquitoes. For instance, during incubation and brooding period, female great tits sleep in the nest, built in cavities that may offer better protection from mosquitoes [79]. Thus, males should be unavoidably more exposed to mosquitoes, which reach a peak of abundance during late spring and early summer [80]. Secondly, susceptibility to *Plasmodium* infection may differ between the two sexes. Males may be more susceptible to parasite infection than females because sex hormones may reduce male’s immunocompetence [5, 81, 82], but also because sex hormones affect disease resistance genes and behaviours [82, 83]. Finally, mosquitoes may prefer males only when being vectors of avian malaria parasite. Some parasites induce remarkable and complex behavioural modifications in their hosts [84]. Lefèvre & Thomas (2008) [85] suggested that parasites may manipulate several phenotypic traits of their vectors to attain a higher transmission probability. Amongst many altered traits, such as biting rate [86], parasites may influence how infected vectors select their vertebrate hosts [53, 87]. According to the ‘‘qualitative manipulation” hypothesis [88], which states that in infected vectors host choice should match the preference of the parasite, it is suggested that the manipulation could occur “at the inter- and/or intra- specific level”, with hosts suitable for the parasite being preferentially chosen [85]. Assuming that males have a weaker immune system than females, parasites should benefit when transmitted to males. Thus, a manipulation of *Cx pipiens* by *Plasmodium* spp. may have evolved and be partly responsible for the sex difference in infection found for *P*. *major*. The most efficient way to test this prediction would be to perform choice experiments with infected vectors.

## Conclusion

We found no sex-biased biting preference of mosquitoes in great tit nestlings. We hypothesize that this is due to the nestlings, as being sexually immature, having undifferentiated body odours. Thus, our results are not in line with the hypothesis that the male-biased *Plasmodium* infection observed in *P*. *major* is caused by an intrinsic preference of *Cx pipiens* mosquitoes for male great tits at this stage of development. A more complex interaction between host, vector and parasite might be a more plausible explanation for the difference in infection found in nature. In particular, we suggest the development of a study that tests adult host preference of uninfected but also infected mosquitoes.

## Supporting information

S1 Fig Schema of the experimental setup. The schema represents the setup in which the host-choice trials were performed.
(TIF)

S1 Table Male-biased *Plasmodium* infection in Swiss great tits. Results of likelihood ratio test performed on generalized linear mixed model fitted using *Plasmodium* infection status as a response variable (binomial error distribution; infected: 1, uninfected:0), sex, age, scaled mass index (MI) and site as explanatory variables and year of sampling as fixed factor. The dataset is the one presented in [51].
(XLSX)

S2 Table Data on host-choice experiment. Each line of the table corresponds to a trial in which a male and a female nestlings coming from the same nest were exposed to mosquito bites during 1 hour.
(XLSX)

S3 Table Results of G-tests of goodness of fit for each site separately, and for all trials per site pooled. Dorigny, Monod and La Praz are the three sites.
(XLSX)
