# Correction: Sex-biased parasitism in vector-borne disease: Vector preference?

**DOI:** 10.1371/journal.pone.0218452

**Published:** 2019-06-11

**Authors:** Camille-Sophie Cozzarolo, Nicolas Sironi, Olivier Glaizot, Romain Pigeault, Philippe Christe

The ORCID iDs are missing for the second, third, fourth, and fifth author. Please see the authors’ respective ORCID iDs here:

- Author Nicolas Sironi’s ORCID iD is: 0000-0002-1321-1718 (https://orcid.org/0000-0002-1321-1718).
- Author Olivier Glaizot’s ORCID iD is: 0000-0001-9116-3355 (https://orcid.org/0000-0001-9116-3355).
- Author Romain Pigeault’s ORCID iD is 0000-0002-8011-4600 (https://orcid.org/0000-0002-8011-4600).
- Author Philippe Christe’s ORCID iD is 0000-0002-8605-7002 (https://orcid.org/0000-0002-8605-7002).
