# Supplementary figures and images for: Sex-biased parasitism in vector-borne disease: Vector preference?
Source: PLoS One. 2019 May 2;14(5):e0216360. doi: 10.1371/journal.pone.0216360 (PMC6497283; doi:10.1371/journal.pone.0216360)

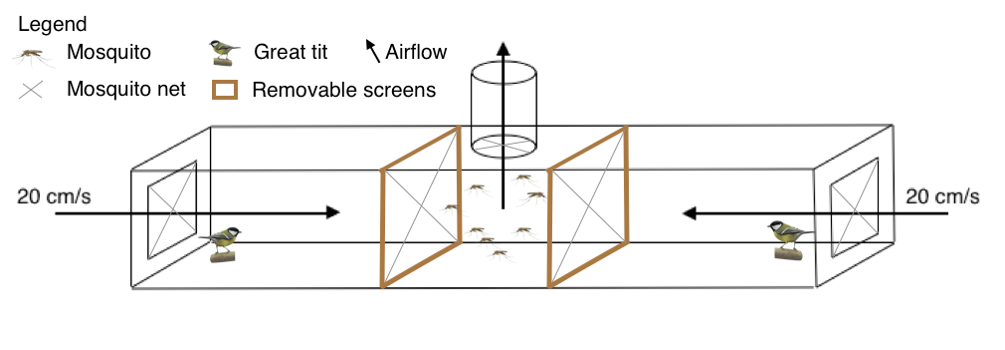

Supplement: S1 Fig — The schema represents the setup in which the host-choice trials were performed. (TIF) [file pone.0216360.s001.tif]
